# Influence of microbiota-driven natural antibodies on dengue transmission

**DOI:** 10.3389/fimmu.2024.1368599

**Published:** 2024-03-15

**Authors:** Alejandra Wu-Chuang, Alejandra Rojas, Cynthia Bernal, Fátima Cardozo, Adriana Valenzuela, Cristina Romero, Lourdes Mateos-Hernández, Alejandro Cabezas-Cruz

**Affiliations:** ^1^ Anses, INRAE, Ecole Nationale Vétérinaire d’Alfort, UMR Virologie, Laboratoire de Santé Animale, Maisons-Alfort, France; ^2^ Universidad Nacional de Asunción, Instituto de Investigaciones en Ciencias de la Salud, San Lorenzo, Paraguay; ^3^ Universidad Nacional de Asunción, Facultad de Ciencias Químicas, San Lorenzo, Paraguay; ^4^ Anses, INRAE, Ecole Nationale Vétérinaire d’Alfort, UMR BIPAR, Laboratoire de Santé Animale, Maisons-Alfort, France

**Keywords:** anti-microbiota vaccines, dengue, microbiome, natural antibodies, alpha-Gal

## Abstract

Dengue has had a significant global health impact, with a dramatic increase in incidence over the past 50 years, affecting more than 100 countries. The absence of a specific treatment or widely applicable vaccine emphasizes the urgent need for innovative strategies. This perspective reevaluates current evidence supporting the concept of dual protection against the dengue virus (DENV) through natural antibodies (NAbs), particularly anti-α-Gal antibodies induced by the host’s gut microbiome (GM). These anti-α-Gal antibodies serve a dual purpose. Firstly, they can directly identify DENV, as mosquito-derived viral particles have been observed to carry α-Gal, thereby providing a safeguard against human infections. Secondly, they possess the potential to impede virus development in the vector by interacting with the vector’s microbiome and triggering infection-refractory states. The intricate interplay between human GM and NAbs on one side and DENV and vector microbiome on the other suggests a novel approach, using NAbs to directly target DENV and simultaneously disrupt vector microbiome to decrease pathogen transmission and vector competence, thereby blocking DENV transmission cycles.

## Introduction

Dengue, also known as break-bone fever, stands as one of the fastest-growing reemerging diseases globally (1). It is a vector-borne illness caused by four serotypes of the dengue virus (DENV), transmitted to humans by *Aedes* mosquitoes. Given their high morbidity and mortality rates, vector-borne diseases, including dengue, pose a significant threat to public health (2–4). The World Health Organization (WHO) reports a dramatic increase in dengue incidence worldwide, with cases soaring 30-fold in the last 50 years (5). Currently, about half of the global population is at risk of dengue, solidifying DENV’s epidemiological significance (6, 7).

Endemic in over 100 countries, dengue primarily affects developing nations in South-East Asia, the Western Pacific, and The Americas (5). For example, Paraguay has faced periodic outbreaks since 1988, when DENV-1 was first detected, followed by the introduction of DENV-2, DENV-3, and DENV-4 in subsequent years (8, 9). Co-circulation of these serotypes has led to significant epidemic outbreaks (9), with the largest reported in 2020, totaling 223,782 suspected cases (8). Dengue, initially confined to sub-tropical and tropical regions, has now emerged in Europe. While initial cases were imported, autochthonous cases have risen since 2010, notably in France (10). In 2023, Europe recorded 105 autochthonous cases, with Italy and France reporting the majority (11). Recently, an autochthonous outbreak of dengue in the Paris Region of France during September–October 2023 was reported (12). The anticipated rise in global dengue cases underscores the ongoing need for research to enhance our understanding of the infection process and develop effective strategies for DENV control. Factors such as expanding mosquito habitats, population growth, climate change, poor urban planning, and insecticide resistance contribute to the increased risk of dengue infections (13–17).

Currently, only one DENV vaccine is available, Dengvaxia^®^, which is restricted to individuals over 9 years old with prior dengue exposure in hyperendemic areas (18). This underscores the need for novel approaches to develop efficient vaccines against DENV.

The microbiome, crucial for human health, plays a central role in host defense against pathogens (19). Natural antibodies (NAbs) induced by the host’s gut microbiome (GM) (20–22), act as a primary defense mechanism (23), targeting various glycans and providing antigen-specific protection against pathogens (22). Notably, anti-Galα1-3Galβ1-4GlcNAc-R (α-Gal) antibodies, the most abundant NAbs in humans, demonstrate a cytotoxic role against α-Gal-expressing pathogens, including viruses like DENV (24). While the direct effect of anti-α-Gal antibodies to DENV in humans remains to be tested, their elevated levels in active DENV infection (25) suggest a potential role in dengue. In addition, recent research indicates that vaccines targeting vector microbiome can induce bacteria-specific antibodies, which, when acquired by blood-sucking vectors, reduce vector fitness and modulate the vector microbiome, reducing pathogen loads (26, 27).

In this *Perspective*, we present the hypothesis that NAbs may confer dual protection against dengue: firstly, by directly protecting against DENV infections in humans, and secondly, by recognizing bacteria in the vector’s microbiome, modulating the microbiome, and reducing vector competence. Understanding the intricate interplay among the host microbiome, NAbs, DENV, and vector microbiome will shed light on the transmission of vector-borne diseases, and holds promise for innovative disease prevention and control approaches.

## Human microbiome and NAbs

The human gut microbiome (GM), an intricate ensemble of bacteria, archaea, protozoans (28), and viruses (29), forms a remarkable ecosystem within our bodies. Housing an astounding population of approximately 100 trillion microorganisms in the gastrointestinal tract alone (30), this microbiome surpasses the total number of cells in the entire human body by almost threefold (31). From a physiological standpoint, this microbial community, constituting around 2% of an adult’s body mass, rivals the size of vital organs such as the brain or liver (32), leading researchers to aptly dub it the “*forgotten*” organ (33, 34). These diverse microorganisms play a pivotal role in numerous processes, contributing to nutrient provision, metabolizing indigestible compounds, defending against opportunistic pathogens, and possessing immunomodulatory properties (35–37). Understanding the intricate interplay between the immune system and GM is crucial for unraveling the association between microbiome and protection to infectious agents.

The immune system’s connection to GM development involves NAbs as key circulating elements, most of which are glycan-specific NAbs (gsNAbs). Despite a limited understanding of the factors governing their repertoire (38), B-1 cells spontaneously produce these antibodies from early life, independent of external immunological stimulation (39, 40). While controversy surrounds the origin, repertoire, and physiological role of antibodies targeting carbohydrate structures (41), the prevailing hypothesis suggests B-1 lymphocytes are stimulated by GM antigenic determinants (41).

In UDP-galactose:β-galactoside-α1-3-galactosyltransferase (α1,3GT)-deficient mice, the gsNAbs repertoire gains diversity between months 1 and 2 of life (21). Remarkably, by month 2, about 41 glycans structures (6% of all glycans structures tested) were highly recognized by at least 60% of α1,3GT-deficient mice sera. Using high-throughput sequencing, the study by Bello-Gil et al. (21) in α1,3GT-deficient mice analyzed GM diversity over 7 months. Associations between gsNAbs and microbial diversity were identified, linking certain bacterial orders to natural anti-glycan antibody development. Microbiome diversity changes correlated with variations in anti-glycan antibody levels and repertoire, suggesting that continuous gut bacterial antigenic stimulation influences antibody repertoire in α1,3GT-deficient mice. In humans, a microchip format glycan array was used to characterize antibody carbohydrate recognition patterns in 106 healthy donors’ sera (42). A glycan library included various blood group antigens, oligosaccharides, glycoproteins, glycolipids, tumor-associated antigens, and bacterial/polysaccharides and lipopolysaccharides. Antibodies in human sera interacted with at least 50 normal human glyco-motifs, revealing surprising features like high antibody binding to specific trisaccharides and sulfated glycans (42). The study unveiled novel binding activities towards certain glycans, such as Galbeta1-3GlcNAc (Le(C)) related glycans and 4’-O-sulfated lactosamine. Notably, the study observed the absence or low binding of antibodies to specific glycans, indicating selective recognition. Antibodies were found to recognize short inner core structures typical for glycolipids and glycoproteins as fragments of larger glycans. Overall, the results suggest diverse and specific binding patterns of antibodies to various glycans in mammals.

Despite uncertainties surrounding the physiological role and origin of circulating anti-glycan NAbs (21), which underscore the complexity of these antibodies, a growing body of evidence describes the functional involvement of anti-glycan antibodies in various immunological mechanisms, both in health and disease (43–46).

**Figure 1:**
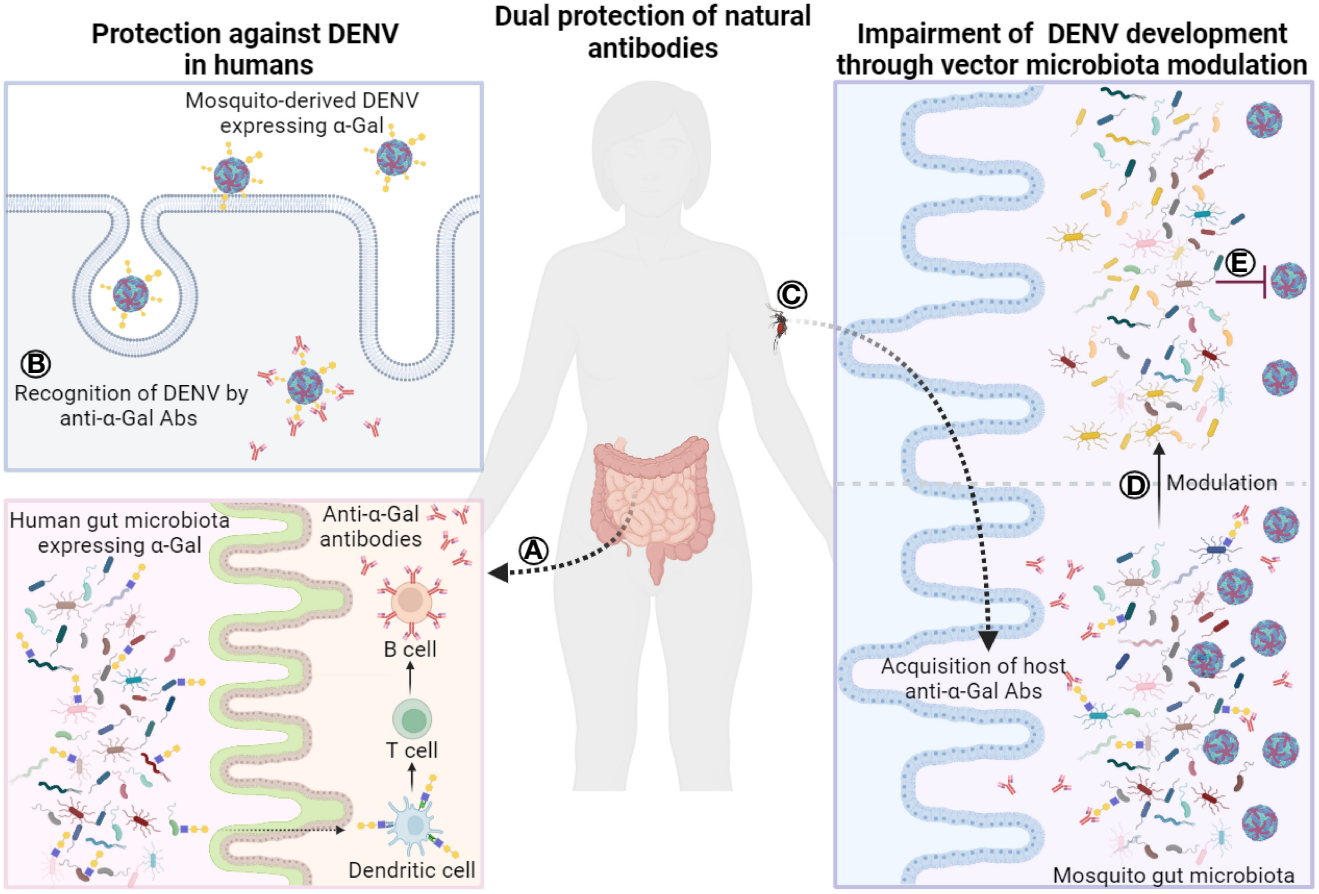
Dual role of natural antibodies. **(A)** Human gut microbiota contains bacteria that express α-Gal epitopes, which in turn, stimulates the production of anti-α-gal antibodies. **(B)** After vector bites, humans acquire mosquito-derived DENV particles containing α-Gal epitopes that are recognized by natural anti-α-Gal antibodies. These antibodies could contribute to the containment or neutralization of the virus limiting its propagation. On the other hand, **(C)** natural anti-α-Gal antibodies can be acquired by mosquitoes during the bloodmeal. These antibodies can reach the gut lumen of mosquitoes and target bacteria that express α-Gal epitopes in mosquito microbiota **(D)** leading to a shift in the bacterial community and structure. Modulation of the mosquito microbiota by natural anti-α-Gal antibodies may **(E)** impair the development of dengue virus in the mosquito limiting the number of viruses to be transmitted. Abs, antibodies. Figure created with BioRender.com.

In this context, it is noteworthy that the inactivation of the UDP-galactose:β-galactoside-α1-3-galactosyltransferase (α1,3GT) gene (*ggta1*), which ablated the expression of α-Gal in old-world monkeys, apes, and humans has bestowed upon this group of primates a unique ability to produce high antibody titers against the glycan α-Gal (47). Notably, among the diverse NAbs produced, those targeting the carbohydrate antigen α-Gal are the most abundant in humans, constituting 1%-5% of circulating immunoglobulins (Ig). GM bacteria induce anti-α-Gal immunoglobulins of the isotypes IgM and IgG, widely expressed in humans (20), fish (IgM) (48–50), and birds (IgM and IgY) (51, 52). At elevated levels, these antibodies provide protection against various infections, including malaria (22, 53), tuberculosis (48–50), ectoparasite infestation (54, 55), and bacterial sepsis (56). Recent research has unveiled the presence of α1,3GT genes, distinct from *ggta1*, in 193 species and strains of bacteria within the human GM (57). Among these bacteria are members of the Enterobacteriaceae family (genus *Escherichia*) and Lactobacillaceae family (genera *Pediococcus* and *Lactobacillus*) (**Figure 1A**).

## NAbs and DENV glycosylation

The potential for glycan-targeting NAbs to confer resistance to DENV in humans is a tantalizing prospect. The diversity of glycans on the surface of DENV plays a crucial role in virus infection and is of significant relevance in the context of neutralizing immune responses. The DENV genome encodes various structural and nonstructural proteins, with the envelope (E) glycoprotein being a key player in viral pathogenesis (58). The E glycoprotein interacts with host cell receptors, initiating the process of virus endocytosis and inducing humoral immune responses, wherein neutralizing antibodies can effectively reduce viral load (59).

While efforts to develop vaccines against DENV primarily focus on stimulating immune responses towards the E glycoprotein (59), the glycans attached to the E glycoprotein remain a complex and not fully understood aspect of DENV (60). N-glycans on the E glycoprotein influence proper protein folding, receptor interactions, and immunogenicity (61). Notably, DENV E glycoprotein has two potential N-linked glycosylation sites at asparagine-67 and asparagine-153 (62), and the sugars added are heterogeneous, consisting of high-mannose and paucimannose glycans (61, 63). These glycans are critical for the virus’s ability to interact with specific receptors, such as dendritic cell-specific ICAM3-grabbing non-integrin (DC-SIGN), found on dendritic cells (DC) in the skin (61, 64).

The interaction between high-mannose glycans on mosquito-derived DENV particles and DC-SIGN facilitates the virus’s entry into immature DC in the skin, a primary target during viral pathogenesis (61). This interaction is crucial for understanding the transmission and infection process of the virus (61). Moreover, the diversity of glycans on the virus surface has been explored as a strategy for designing carbohydrate-based antiviral agents, as demonstrated with oligomannosides inhibiting DC-SIGN-mediated human immunodeficiency virus (HIV)-1 infection (65).

Despite progress, the analytical challenges in characterizing the precise structures of glycans on the DENV E glycoprotein surface persist due to their heterogeneity and variable glycosylation site utilization (66). Recent technological advances, such as lectin microarray and mass spectrometry, have become essential tools in resolving this heterogeneity (67, 68). Studies have identified a wide range of N-linked glycans on DENV, including mannose, N-acetylgalactosamine (GalNAc), N-acetylglucosamine (GlcNAc), fucose, and sialic acid (69). High-mannose-type N-linked oligosaccharides and galactosylation were found to be major structures, highlighting the complexity and diversity of glycans on the DENV surface (69).

The importance of glycans is further emphasized by their involvement in viral morphogenesis, infectivity, and tropism (62, 70). The heterogeneity of glycans on DENV E proteins derived from insect and mammalian cells underscores the need for comprehensive studies to characterize the N-linked sugar structures at each potential glycosylation site (62).

Detailed information obtained through lectin array and mass spectrometry reveals a high heterogeneity in N-glycans on DENV-2, including mannose branching, high-mannose-type N-glycans, galactosylation, bisecting GlcNAc, and sialylation (71). These diverse glycan structures are crucial for understanding the virus-host interactions, particularly with DC-SIGN on DC cells (72). Computational docking experiments suggest specific glycans on the DENV-2 surface as potential ligands for DC-SIGN, further illuminating the intricate interplay between viral glycans and host receptors (73).

The structural features of N-glycan structures on the surface of mature DENV-2 derived from *Aedes albopictus* cells (24) may play a crucial role in interactions with the immune system, particularly with glycan-specific natural antibodies found in α1,3GT-deficient mice (21). The presence of common glycan motifs such as galactose (Gal), GalNAc, fucose, GlcNAc, and sialic acid in both DENV-2 surface and antibody specificities suggests a potential for recognition by glycan-specific antibodies. For instance, the blood group antigen structures, high-mannose features, and terminal GalNAc residues found on DENV-2 may be targets for NAbs in α1,3GT-deficient mice, a model for human α-Gal immunity. Additionally, the recognition of sulfation modifications and complex oligosaccharide structures by NAbs (21) could further contribute to the recognition of DENV particles.

The interaction between these NAbs and DENV-2 glycans, particularly after mosquito transmission, may influence the immune response to the virus. The recognition of specific glycan motifs on DENV-2 by NAbs could potentially modulate immune reactions and contribute to the overall understanding of the host-pathogen dynamics in DENV-2 transmission via *Ae. albopictus* mosquitoes. Further experimental studies would be necessary to validate and elucidate the detailed mechanisms underlying the interactions between DENV glycans and NAbs in the context of mosquito transmission.

Notably, the glycan profile of DENV-2 includes the presence of α-Gal structures (24). This α-Gal motif was associated with signals from lectins, such as *Amaranthus caudatus* agglutinin (ACA), *Ricinus communis* agglutinin (RCA) 120, *Euonymus europaeus* lectin (EEL), *Bandeirea simplicifolia* agglutinin (BS)-I, *Bauhinia purpurea* lectin (BPL), and *Psophocarpus tetragonolobus* lectin (PTL)-II (24). The source of α-Gal in DENV is proposed to be the mosquito salivary glands (22), as it is absent in DENV amplified in humans after the initial wave of infection. Interestingly, the enzyme responsible for α-Gal production, α1,3GT, has not been identified in arthropod genomes, suggesting the involvement of other galactosyltransferases in the synthesis of this glycan in arthropods (74–76). Indeed, genes like alpha-1,4-galactosyltransferase, beta-1,3-galactosyltransferase, and alpha-1,4-galactosyltransferase have been reported in ticks (75–77). Moreover, orthologs of some of these genes, exhibiting α1,3GT activity in ticks (76), have been identified in mosquitoes (74). Importantly, the existence of Gal and α-Gal structures on the surface of DENV-2 raises the possibility that they may serve as targets for anti-α-Gal NAbs in humans (**Figure 1B**).

Patients with active DENV infections exhibit significantly elevated levels of anti-α-Gal IgM and IgG (25), suggesting a plausible involvement of these antibodies in the dengue infection process. In the examined population, dengue IgM positive patients demonstrated significantly higher levels of anti-α-Gal IgM (25). This suggests that IgM antibodies targeting α-Gal play a role in the early immune response during DENV infection. Specifically, anti-α-Gal IgM may be involved in the immediate recognition and neutralization of the virus inoculated with mosquito saliva into dermal and epidermal cells. This initial response could contribute to the containment of the virus at the site of entry, potentially limiting its dissemination and preventing systemic infection. On the other hand, the study reveals a correlation between anti-α-Gal IgG levels and severe dengue symptoms (25). The delayed production of anti-α-Gal IgG, occurring after the expected first wave of viremia, suggests that this antibody isotype may play a distinct role in the later stages of infection. Unlike IgM, IgG is associated with a more prolonged immune response and is capable of providing long-term protection. The increased levels of anti-α-Gal IgG may function as a protective antibody, potentially limiting additional DENV transmission through mosquito bites. This could be especially relevant in dengue hyperendemic areas, where repeated exposure to the virus is common. However, the study also found anti-α-Gal IgG to be correlated with severe dengue symptoms, suggesting a dual role in protection and disease progression. The potential roles of anti-α-Gal antibodies present a dichotomy, with the antibodies possibly acting protectively against DENV infections and/or contributing to antibody-dependent enhancement (ADE) in DENV infection (78). The protective and ADE affects may not be mutually exclusive, and concentration-dependent effects of anti-α-Gal antibodies, coupled with population studies, are essential for a conclusive understanding of this phenomenon (25).

Several studies support the notion that anti-α-Gal antibodies can act as neutralization antibodies against various pathogens, including *Plasmodium* spp (22, 53), *Mycobacterium* (48–50), *Leishmania* spp (79), and *Trypanosoma cruzi* (80). Geographical variations in anti-α-Gal antibodies were observed (25, 81), suggesting an association with the progression of dengue disease (25). The study also indicates age-related correlations with anti-α-Gal antibody levels (25), introducing complexities that warrant further investigation. Overall, the evidence provides valuable insights into the roles of anti-α-Gal antibodies in the context of DENV infection, emphasizing the need for comprehensive and context-specific evaluations of their functions and implications.

## DENV and vector microbiome

The interaction between mosquitoes and the DENV is complex, involving various factors that influence vector competence and, consequently, the transmission of the virus. One crucial element is the mosquito microbiome, particularly the midgut microbiome, which plays a significant role in shaping the vector’s ability to transmit pathogens (82, 83). Studies have shown that the microbiome in the mosquito gut influences key physiological processes related to pathogen transmission (84, 85). For instance, the depletion of gut bacteria renders mosquitoes more susceptible to DENV, and the reintroduction of specific bacterial species results in decreased viral load (86–88). In contrast, the colonization of mosquito larvae with *Salmonella* sp. increases DENV infection in adult mosquitoes compared to those colonized with a mixture of different bacteria in the family Enterobacteriaceae (89). Furthermore, altering the mosquito microbial community through exposure of larvae to a pathogenic *Bacillus thuringiensis* (90), enhances adult mosquito susceptibility to DENV but not to Chikungunya virus (CHIKV) (91). These findings suggest pathogen-specific interactions within the mosquito microbiome.

The midgut microbiome is presumed to exert antiviral activity through both direct and indirect mechanisms, involving the activation of innate antiviral responses and the production of antimicrobial peptides (AMPs) (87, 88). Additionally, specific bacteria, such as *Chromobacterium* sp. isolated from the mosquito midgut, produce compounds that directly inhibit DENV attachment and invasion within host cells (92), while *Serratia odorifera* bacteria can increase DENV-2 infections in *Ae. aegypti* (93). In addition, studies on genetically selected DENV-resistant and -susceptible *Ae. aegypti* strains suggest that certain bacterial genera may serve as biomarkers for vector competence (94, 95). The impact of the midgut microbiome on vector competence for DENV-2 is further highlighted by findings showing that B vitamin provisioning or introduction of B vitamin-autotrophic bacteria increases viral replication (96).

The impact of *Wolbachia* infections on vector competence for DENV is a notable aspect of mosquito microbiome research. *Wolbachia*, a type of endosymbiont, can block the development of pathogens such as DENV and Zika virus (ZIKV) in specific host–pathogen–*Wolbachia* combinations (97–103). Control strategies based on *Wolbachia* include the use of large numbers of *Wolbachia*-infected males for population suppression or elimination through incompatible insect technique (IIT), causing a population crash (104, 105). Alternatively, spreading a pathogen-blocking *Wolbachia* strain could replace local permissive natural vectors with refractory insects, and this strategy is being evaluated in field trials to reduce DENV transmission by *Ae. aegypti* mosquitoes in various regions (106). Stable *Wolbachia* infections have been detected in natural Anopheles populations, challenging the previous belief that anopheline mosquitoes are resistant to colonization by these bacteria (107, 108). Interestingly, the microbiome composition of *Ae. aegypti* is not critical for *Wolbachia*-mediated inhibition of DENV (109).

The interaction between DENV and its mosquito vectors is a multifaceted process intricately influenced by the mosquito microbiome, especially within the midgut. This microbiome is vital for determining the mosquito’s vector competence, influencing both susceptibility and resistance to DENV through mechanisms such as modulation of the immune response, production of antimicrobial peptides, and direct inhibition of viral processes. The dynamic interplay between different bacterial species within the mosquito gut and their pathogen-specific effects on DENV transmission highlights the potential for microbiome-targeted interventions in controlling dengue spread. Future research should aim to deepen our understanding of these complex interactions and explore innovative strategies for manipulating the mosquito microbiome.

## Disrupting vector microbiome with NAbs to block DENV

Beyond directly targeting viral particles, another avenue for defense against DENV involves directing NAbs towards vector microbiota (**Figure 1C**). To interfere with DENV development within mosquitoes, we can draw parallels from recent advancements in tick-borne pathogens (26) and avian malaria (27). Ticks, akin to mosquitoes, transmit medically significant pathogens, such as *Borrelia afzelii* causing Lyme borreliosis (110). Targeting keystone taxa of tick microbiome through anti-microbiota vaccines has proven effective in altering tick feeding (54) and influencing the taxonomic and functional profiles of bacterial communities (111). The success of this approach is highlighted by recent findings in tick research, where perturbation of tick microbiome resulted in a lower load of the pathogen *B. afzelii* (26).

Applying a similar approach to mosquitoes, we can develop anti-microbiota vaccines that elicit host antibodies against specific bacteria or their products, disrupting the delicate balance in the mosquito midgut microbiome (27, 112) (**Figure 1D**). This strategy finds support in recent success with anti- microbiome vaccines applied to mosquitoes in avian malaria research (27). For instance, when *Culex quinquefasciatus* mosquitoes fed on *Escherichia coli*-immunized canaries, there were deviations from the typical microbiome changes induced by the malaria parasite *Plasmodium relictum* (27). This resulted in reduced pathogen levels in both midguts (oocyst) and salivary glands (sporozoites) of the vector (27). The application of anti-microbiota vaccines targeting keystone taxa of mosquito microbiome, as exemplified by Enterobacteriaceae, can be harnessed to alter vector feeding behavior and modulate the taxonomic and functional profiles of the mosquito microbiome. Drawing from the successes observed in avian malaria and Lyme Borrelia control in mosquitoes and ticks, respectively, through anti-microbiota vaccination, we could explore a similar approach to disrupt DENV within mosquitoes. This involves leveraging the modulation of the vector’s midgut microbiome by host antibodies to significantly impede the development of the pathogen (**Figure 1E**).

Moreover, a hierarchical shift in the *Ae. albopictus* microbiome caused by an anti-microbiota vaccine, leading to increased fecundity and egg-hatching rate in female mosquitoes, provides additional insights (112). The impact of host antibodies on mosquito microbiota and life traits, exemplified by the significant increase in fecundity and egg-hatching rate in mosquitoes fed with blood of *Chryseobacterium*-immunized rabbits (112), suggests that specific alterations to the microbiota can influence mosquito fitness and reproduction. This highlights the potential of targeted vaccines to induce infection-refractory states in the mosquito microbiome (113), thereby disrupting the life cycle of vector-borne pathogens like DENV. As network analyses revealed alterations in the hierarchical organization of bacterial communities, this information can guide the rational design of anti-microbiota vaccines to reduce vector fitness and block pathogen transmission effectively. In summary, leveraging the knowledge gained from tick and mosquito studies, anti-microbiota vaccines represent a promising tool for disrupting the development of tick-borne and mosquito-borne pathogens within their respective vectors.

## Discussion

The alarming surge in dengue cases globally (1), especially in hyperendemic areas like Paraguay, necessitates innovative approaches to combat this reemerging disease. The lack of specific treatments or widely applicable vaccines underscores the urgency for novel strategies. Leveraging the intricate interplay between human microbiome, NAbs, and vector microbiome presents a promising frontier.

The unique ability of NAbs, particularly those targeting α-Gal, to recognize and combat pathogens provides a foundation for exploration. While the role of these antibodies face to dengue virus infection is still unknown and needs further investigation, we hypothesize that anti-α-Gal antibodies may limit the dissemination of DENV by complement-mediated and antibody-mediated opsonization since it was found that mosquito-derived DENV particles carry α-Gal epitopes on its surface (114). Neutralization of viruses by anti-α-Gal antibodies could also be another plausible mechanism of inhibition of virus propagation. It has been found that swine serum supplemented with exogenous anti-α-Gal antibodies neutralized Pseudorabies virus grown in a porcine kidney cell line (115). Anti-α-Gal antibodies in DENV-infected humans may exert a dual mechanism of inhibition of DENV by neutralization and lysis. However, this hypothesis remains to be tested. If these antibodies indeed play a role in resisting DENV, harnessing them could be a groundbreaking strategy for developing effective and innovative vaccines (81, 116). Furthermore, the modulation of vector microbiome, inspired by the success of *Wolbachia* in impeding pathogen development (83), and microbiota-targeted vaccines (26, 27), introduces a dual-pronged approach. Directly targeting viral particles in humans and interfering with the virus’s lifecycle within mosquitoes could significantly mitigate DENV transmission.

The emergence of dengue in non-endemic regions like Europe emphasizes the need for continuous research and innovation. Autochthonous cases underscore the adaptability of the virus and the urgency of devising global strategies against its spread. While the implementation of *Wolbachia* in field trials is a promising step, further research into identifying and understanding human microbiome bacteria that induce protective NAbs is essential. This knowledge could provide a foundation for tailored interventions and vaccine development.

Incorporating anti-microbiota vaccination strategies into the fight against DENV represents a novel and innovative approach that leverages the host’s immune response to disrupt the vector microbiome, thereby impeding the mosquito’s ability to transmit the virus. Target populations for receiving these vaccinations would ideally include individuals in both endemic and non-endemic regions who are at high risk of exposure to DENV. In endemic areas, vaccination efforts could focus on communities with high transmission rates to reduce the overall burden of disease and interrupt the transmission cycle. In non-endemic regions, vaccinations could be targeted toward travelers to endemic areas, healthcare workers, and military personnel who may be exposed to DENV through their activities. The timing of vaccinations should be strategically planned to coincide with periods of increased mosquito activity and before the peak dengue season in endemic regions to maximize the protective effects of the vaccine. This preemptive approach would allow the host immune system sufficient time to develop antibodies against the targeted microbiota components before exposure to DENV-carrying mosquitoes.

While the concept of altering the vector microbiome by vaccinating hosts presents a promising avenue for controlling DENV transmission, it is crucial to underscore that this strategy is still in its developmental stages. Extensive research is needed to ascertain the efficacy and safety of these vaccines. Critical considerations include determining the specific bacterial targets within the mosquito microbiome that, when disrupted, would most effectively reduce vector competence without adversely affecting the host. Additionally, it is imperative to evaluate the long-term effects of such vaccinations on both the host’s microbiome and ecosystem biodiversity to ensure that they do not inadvertently cause harm. As we explore the potential of anti-microbiota vaccinations, it is essential to maintain a cautious and evidence-based approach. This involves conducting rigorous preclinical and clinical trials to fully understand the implications of these vaccines on human health and mosquito populations. The development of these vaccines should be complemented by ongoing surveillance of vector populations and continued research into the complex interactions between hosts, vectors, and pathogens. This comprehensive strategy will ensure that anti-microbiota vaccinations, once proven safe and effective, can be seamlessly integrated into existing vector control and disease prevention frameworks, offering a novel tool in the global effort to combat DENV and other vector-borne diseases.

In conclusion, the exploration of NAbs induced by human microbiome as potential weapons against DENV opens up exciting avenues for research and intervention. By strategically directing these antibodies to target viral particles and vector microbiome, we may unlock new possibilities in the fight against dengue. The urgency of the situation demands collaborative efforts to bridge the gap between research findings and practical, scalable solutions, ultimately paving the way for a dengue-resistant future.

## Author contributions

AW-C: Writing – review & editing, Writing – original draft, Conceptualization, Visualization. AR: Writing – review & editing. CB: Writing – review & editing. FC: Writing – review & editing. AV: Writing – review & editing. CR: Writing – review & editing. LM-H: Writing – review & editing. AC-C: Writing – review & editing, Writing – original draft, Conceptualization.
